# Medication coaching program for patients with minor stroke or TIA: A pilot study

**DOI:** 10.1186/1471-2458-12-549

**Published:** 2012-07-25

**Authors:** Elizabeth G Sides, Louise O Zimmer, Leslie Wilson, Wenqin Pan, DaiWai M Olson, Eric D Peterson, Cheryl Bushnell

**Affiliations:** 1 Department of Neurology, Wake Forest School of Medicine, Winston-Salem, NC, USA; 2 Duke Clinical Research Institute, Duke University School of Medicine, Durham, NC, USA; 3 Department of Medicine, Division of Neurology, Duke University Medical Center, Durham, NC, USA

**Keywords:** Stroke, Transitions, Prevention, Patient education

## Abstract

**Background:**

Patients who are hospitalized with a first or recurrent stroke often are discharged with new medications or adjustment to the doses of pre-admission medications, which can be confusing and pose safety issues if misunderstood. The purpose of this pilot study was to assess the feasibility of medication coaching via telephone after discharge in patients with stroke.

**Methods:**

Two-arm pilot study of a medication coaching program with 30 patients (20 intervention, 10 control). Consecutive patients admitted with stroke or TIA with at least 2 medications changed between admission and discharge were included. The medication coach contacted intervention arm patients post-discharge via phone call to discuss risk factors, review medications and triage patients’ questions to a stroke nurse and/or pharmacist. Intervention and control participants were contacted at 3 months for outcomes. The main outcomes were feasibility (appropriateness of script, ability to reach participants, and provide requested information) and participant evaluation of medication coaching.

**Results:**

The median lengths of the coaching and follow-up calls with requested answers to these questions were 27 minutes and 12 minutes, respectively, and participant evaluations of the coaching were positive. The intervention participants were more likely to have seen their primary care provider than were control participants by 3 months post discharge.

**Conclusions:**

This medication coaching study executed early after discharge demonstrated feasibility of coaching and educating stroke patients with a trained coach. Results from our small pilot showed a possible trend towards improved appointment-keeping with primary care providers in those who received coaching.

## Background

Stroke is a common and devastating disease that affects almost 800,000 people in the US each year and is a leading cause of serious long-term disability [1]. Approximately 610,000 of these strokes are first attacks, and 185,000 are recurrent attacks [1]. The transition to home after an acute stroke hospitalization requires that patients make substantial adjustments to learn how to cope with their condition, their medications and, potentially, new disabilities within a short timeframe. Early supported discharge for stroke patients shows promise towards facilitating the transition from hospital to home, although this approach is more commonly utilized in Europe [2]. Frequently, however, stroke survivors and their caregivers are unprepared for this new role: they may not know the patient’s discharge diagnosis, may have difficulty understanding complex medication regimens and be unsure who to contact with questions about their condition or prescribed medications [3-5]. As expected, patients have reported dissatisfaction with the quality and quantity of information provided by health professionals during their hospital stay [6].

The kind of information required by patients varies depending upon the care setting and their health care needs [7-9]. In-depth qualitative interviews of stroke patients found that in the first month post-discharge, patients valued individualized information about causes of their stroke and prevention of recurrence; the significance of symptoms and how they should be managed; and information on medications, such as how to obtain refills or even if refills are needed [6]. The provision of information, tools and structured support in the transition to home has been shown to improve patients’ understanding of their diagnoses and medications, increase patients’ confidence in their ability to manage their condition, decrease patient anxiety, improve health outcomes and reduce hospital re-admissions [3,5,7,9-15].

Key elements of effective care transitions include assistance with managing medications; written material that includes critical medical information and a list of the patient’s medications, information on warning signs and adverse events and instructions on how they should be managed, including who to contact; and encouragement to follow-up with primary or specialty care in a timely manner [5,6,10,16]. One study demonstrated that patient counseling with a single phone call shortly after hospital discharge resulted in a statistically significant decrease in preventable adverse drug events and significantly higher satisfaction with medication instruction [17].

Our goal is the development of a program to support patients in their transition to home after hospitalization for stroke or transient ischemic attack (TIA). We designed a pilot study to evaluate and refine a medication coaching program before it was implemented as hospital-wide standard of care. The objectives were: a) assess the feasibility of the intervention in terms of the appropriateness of script, ability to reach participants, and provide the requested information, and b) to assess the preliminary impact of the intervention on medication knowledge, medication persistence, and appointment-keeping.

## Methods

### Design overview

**Figure 1:**
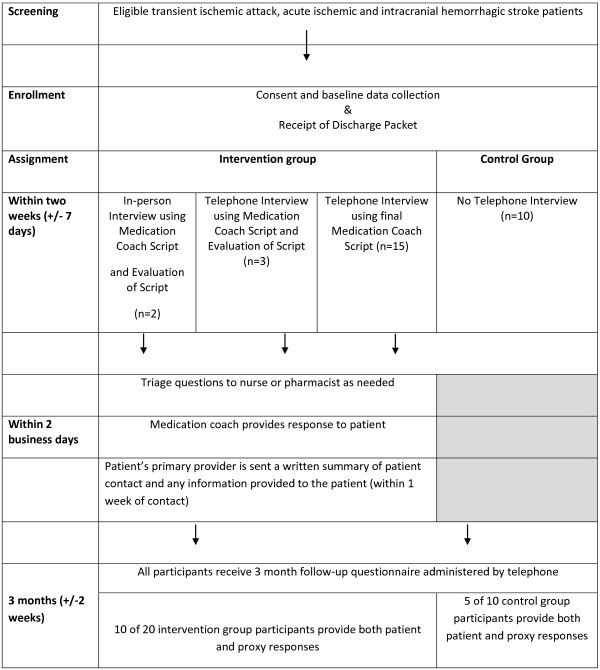
The medication coaching study design.

We performed a two-arm pilot study of 30 patients at Wake Forest Baptist Medical Center (WFBMC) admitted with ischemic or hemorrhagic stroke or TIA who had at least 2 medications changed between admission and discharge and who were discharged home. Figure 1 provides an overview of the study design.

**Figure 2:**
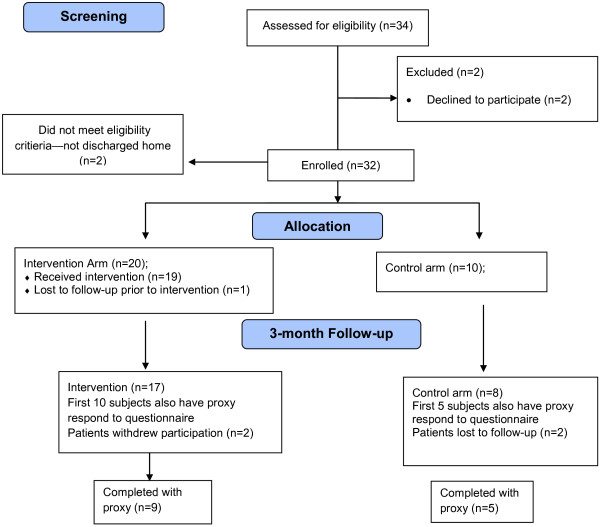
Flow diagram of study enrollment, group assignment, and follow-up at 3 months.

With daily review of all neurology hospital admissions, consecutive, potential participants who met the eligibility criteria were visited by the study coordinator who explained the study and reviewed the consent form. Each subject who agreed to participate signed a consent form. They were also asked to provide a phone number where they could be reached once they were released from hospital as well as that of a close friend or relative who would be familiar with their whereabouts. The study was approved by the Institutional Review Board of both the study institution and the data coordinating center (Duke Clinical Research Institute) prior to the enrollment of subjects. Figure 2 shows the flow from screening to follow-up.

### Study procedures

Prior to hospital discharge, all participants received an information packet containing materials on when to call 911 or their physician and/or pharmacist; general lifestyle information for stroke prevention; a check list of their personal risk factors and additional information about each; and a list of their current medications, what they were for and dosage. The medication coach reviewed the contents of the packet with each participant prior to discharge.

The first 20 participants were intended to receive the intervention, which consisted of a telephone contact from a medication coach within the first 2 weeks after discharge and included general information about stroke, the importance of preventing another stroke, modifiable risk factors and the importance of adhering to prescribed medications. In addition, the medication coach reviewed each stroke prevention medication on the participant’s discharge list and asked whether he or she was still taking the medication, and if not, why not. Finally, the coach assessed the participant’s understanding of each medication’s purpose, how to take it, refill it, and its side effects. Participants were also asked if they had any specific questions about their medications or stroke recovery. The coach triaged medication-related questions to a pharmacist and stroke-related questions to a stroke nurse, then compiled responses from these clinicians, and called the subject back with the information. Compiled responses were also summarized in written form and sent to the participant’s primary care provider with a cover letter explaining the patient’s participation in the program. Detailed or lengthy answers to participants’ questions were summarized on a 7^th^ grade reading level and sent to each participant in a follow-up letter from the medication coach.

Participants in the control arm (n = 10) were not called by the medication coach. All participants were contacted at 3 months to assess medication use, appointment keeping, functional status, and resource utilization.

### Coaching script development

The medication coaching script and the discharge packet information were written at a 7^th^ grade reading level. The first two participants had their 2-week medication coaching session as an in-person interview during which they also provided feedback on the process. The next 3 participants were contacted via telephone for the coaching call and they also gave feedback. To aid in the development of the medication coaching “script,” these first 5 participants evaluated the length, content and usefulness of the interaction and the information provided with a staff member from the data coordinating center via telephone. After the initial 5 interviews were completed, the study team made final revisions to the study forms and scripts. The next 15 participants were contacted via telephone approximately 2 weeks after hospital discharge for their interviews using the revised script and forms. The intervention was designed to be supplemental to the care, education and information provided by the stroke unit physicians, nurses and other medical staff members.

### Data collection

Baseline data were obtained from a combination of medical record review and participant interview prior to discharge and included: pre-admission stroke prevention medications; history of stroke risk factors; family history of stroke; NIHSS on admission; functional status; demographic and socioeconomic information; specific diagnosis; hospital course; laboratory values; perception of patient-provider communication; patient ability to care for self; perception of health status (EuroQOL-5-D or EQ-5D)[18]; depression status (Patient Health Questionnaire-8 or PHQ-8)[19]; complications and discharge stroke prevention medications.

Interviewers from the data coordinating center conducted the 3-month telephone interviews (not the medication coach), and asked about current stroke prevention medications; medications that were stopped and reason for stoppage; functional status; demographic and socioeconomic information; current smoking status; Care Transitions Measure (CTM-3) [20]; patient-provider communication; patient’s ability to care for self; medication tracking/assistance; patient satisfaction with hospital treatment, care and information provided; perceived burden of medical costs; health insurance status; current status of stroke/stroke recovery; functional status (modified Rankin score); perception of health state (EQ-5D); depression status (PHQ-8); hospitalizations and ED visits since discharge; and post-discharge rehabilitation utilization.

In addition to the 3-month participant interviews, for 10 of the 20 intervention group participants and 5 of the 10 control group participants, interviews were conducted with a proxy designated by the participant at the initial hospital interview. Proxy interviews enabled us to collect follow-up data on 4 participants we were unable to contact and all proxies provided additional information related to medication persistence and participants’ status at 3 months.

### Data analysis

The primary outcomes of this pilot study are: 1) feasibility of the intervention (ability to reach participants, number of attempts, and time spent on the telephone), and 2) participants’ evaluation of the medication coaching script and its implementation.

The secondary outcomes are: 1) impact of intervention on medication persistence, knowledge, and appointment-keeping; 2) clinical and functional outcomes (including depression) at 3 months, and 3) resource utilization (re-hospitalizations and Emergency Department visits) at 3 months.

Descriptive statistics included counts with percentages for discrete variables and medians with interquartile ranges for continuous variables. Comparisons were performed according to treatment assignments (intervention arm versus control arm).

We compared overall persistence between groups as an all or none variable (i.e., subjects who remained on all discharge medication classes at the 3-month follow-up were considered persistent), whereas subjects who stopped at least one class of medication prescribed at discharge were “non-persistent” overall regardless of the reason for stopping a medication. We also summarized persistence at the class level.

## Results

Of 32 enrolled participants, two patients were ineligible because they were not discharged to home. One intervention patient was never reached to perform the medication coaching and was unavailable for follow-up; thus, there were 19 patients in the intervention group and 10 in the control group. Characteristics in the two groups were similar, although those in the control arm were more likely to be white, to be slightly younger and to report having health insurance (Table 1). A greater percentage of subjects in the intervention arm had a stroke as opposed to a TIA. Intervention subjects were discharged with a somewhat higher median number of stroke secondary prevention medications (median = 5 medications vs control median = 4).

**Table 1:** Baseline Characteristics of Enrolled Subjects

**Characteristics**	**Overall (n = 29)**	**Intervention Arm (n = 19)**	**Control Arm (n = 10)**
Female	12 (41.4 %)	8 (42.1 %)	4 (40.0 %)
Median age (IQR)	61 (52–69)	61(55–70)	59 (47–67)
Race			
African American	13 (44.8)	10 (52.6)	3 (30)
White	16 (55.2)	9 (47.4)	7 (70)
NIHSS, median (IQR)	1(1–3)	2 (1–4)	1 (1–2)
Stroke type			
Ischemic stroke	24 (82.2)	17 (89.5)	7 (70.0)
TIA	4 (13.8)	2 (10.5)	2 (20.0)
ICH	1 (3.5)	0	1 (10.0)
Medical history (or risk factors)			
Prior stroke/TIA	10 (34.5)	7 (36.8)	3 (30.0)
Hypertension	22 (75.9)	16 (84.2)	6 (60.0)
Hyperlipidemia	20 (71.4)	14 (73.7)	6 (66.7)
Smoker	14 (48.3)	8 (42.1)	6 (60.0)
Diabetes	6 (20.7)	3 (15.8)	3 (30.0)
Atrial fibrillation	3 (10.3)	2 (10.5)	1 (10.0)
Number of discharge meds, median (IQR)	4 (3–6)	5 (3–6)	4 (2–6)
Health Insurance			
Yes	21 (72.4)	13 (68.4)	8 (80)
No	8 (27.6)	6 (31.6)	2 (20)
Health Insurance Type			
Public	7 (33.3)	4 (30.8)	3 (37.5)
Private	9 (42.9)	6 (46.2)	3 (37.5)
Public and private	5 (23.8)	3 (23.1)	2 (25.0)
Income meets basic needs			
More than adequately	4 (14.3)	3 (15.8)	1 (10.0)
Adequately	7 (25.0)	6 (31.6)	1 (10.0)
Somewhat	12 (42.9)	7 (36.8)	5 (50.0)
Not at all	5 (17.9)	3 (15.6)	2 (20.0)
Missing	1 (3.5)	0	1 (10.0)

IQR = interquartile range; NIHSS = National Institutes of Health Stroke Scale; TIA = transient ischemic attack; ICH = intracerebral hemorrhage; PCP = primary care provider.

### Medication coaching script refinements and evaluations

After the first patient, concerns were raised regarding the validity of using only yes/no responses when asking if patients understand their medications. For better assessment of participant competence and understanding, the questions were changed to an open-ended format. This change also enabled more appropriate teaching moments during the call. Evaluations from the first 5 participants regarding the medication coaching telephone calls were favorable (Table 2).

**Table 2:** Evaluation of medication coaching intervention (first 5 participants)

	**Strongly Agree**	**Agree**	**Comments**
Information we provided about stroke was helpful to you.	40%	60%	‘wanted information on what to expect in future,’ ‘more about prevention’
Information we provided about your medications was helpful to you.	60%	40%	‘explained purpose of medications and side effects’
Contact was an appropriate length.	80%	20%	
Interviewer talked in a way that was easily understood.	100%		
If you requested additional information, were your questions answered to your satisfaction when you were called back?	100% (n = 3)		
Other information that would be useful now that you’re home recovering from your stroke?			‘stress prevention and nutrition,’ ‘covered everything I could think to ask’

### Assessment of medication knowledge during coaching calls

During the medication coaching contact, 7 out of 19 intervention group participants were able to identify why they were taking all of their medications and an additional 9 could state the reason for taking 50% or more of their medications. Likewise, most could explain how to refill their medications. However, none could identify one side effect for all prescribed medications and 5 of the 19 were unable to list any side effects. All participants taking warfarin (n =4) were able to identify at least one side effect and knew that they required regular blood tests to determine its effectiveness.

The types of questions that were asked during the coaching intervention call were similar among the intervention participants, and frequently concerned why the stroke occurred even while taking prevention medications, and how to prevent another stroke. One 57 year-old participant asked why he was still having fatigue after the stroke and whether it was due to his medications. The pharmacist’s response was that this was quite possibly due to low blood pressure, and suggested the patient take one of his three blood pressure medications at bedtime and the other two in the morning to see if this helps the fatigue.

### Follow-up calls and logistics

Fourteen of the 19 intervention patients required more than one phone call to conduct the coaching intervention, even within the two weeks after discharge. The median number of calls required to complete the medication coaching call was 2 (range 1–9). The median lengths of the coaching and follow-up calls with requested answers to questions were 27 minutes and 12 minutes, respectively.

Four participants out of 29 could not be contacted for the 3-month interview, even after multiple attempts. Because of the difficulty in reaching patients and proxies, 20 out of 37 of these interviews were completed after the 3 month target time window. The reasons for not reaching patients included: disconnected numbers, people out of country/out of state, and 2 patients were living with various relatives and moving frequently. For 2 other patients in the control arm, interviewers were only able to interview the proxy and not the patient.

The number of attempts made to reach an individual patient or proxy for their 3-month follow-up interview ranged from 1 to 30, and there were no differences between intervention and control participants. We were unable to collect 3-month follow-up information for 2 participants from the intervention arm (10.3% lost-to-follow-up but proxy data were obtained).

### Three month outcomes

For those successfully contacted at 3 months and able to provide self-reported outcomes, we found little difference between the two groups in reported levels of knowledge or understanding about medications or stroke (Table 3). More of the intervention participants knew what to do if problems or symptoms continued or worsened (93.8%) compared to controls (77.8%). A larger proportion of intervention participants had seen their primary care provider between discharge and 3 months than those in the control group (93.8% vs. 60% in the controls; p = 0.055). In addition, we found trends towards lower depression (PHQ-8) severity scores in the intervention group (median = 5.50 vs. 10.5 in controls; p = 0.080), higher reported health status (EQ-5D) scores (median 0.80 vs. 0.68), and lesser disability (mRS =1.5 in cases vs. 2.5 among controls.)

**Table 3:** Three month outcomes for intervention and control participants

**Characteristics**	**Overall (n = 26)**	**Intervention Arm (n = 16)**	**Control Arm (n = 10)**	p-value**
Has method for tracking medications, N (%)	18 (69.2)	11 (68.8)	7 (70.0)	1.000
Understand how to take medications, N (%)	26 (100.0)	16 (100.0)	10 (100.0)	N/A
Understand why taking medications, N (%)	26 (100.0)	16 (100.0)	10 (100.0)	N/A
Understand side effects, N (%)	16 (61.5)	10 (62.5)	6 (60.0)	1.000
Know who to call if run out of meds, N (%)	26 (100.0)	16 (100.0)	10 (100.0)	N/A
Know what to expect with your health/illness in the future, N (%)	19 (73.1)	12 (75.0)	7 (70.0)	1.000
Know what to do if problems/symptoms continued or worsened, N (%)	22 (88.0)	15 (93.8)	7 (77.8)	0.530
Appointment with PCP since stroke, N (%)	21 (80.8)	15 (93.8)	6 (60.0)	0.055
PHQ-8 at 3 months, median (IQR)	8 (3.0-13.0)	5.50 (0.5-10.5)	10.5 (7.0-21.0)	0.080
EQ5D at 3 months, median (IQR)	0.8 (0.64-0.87)	0.80 (0.69-0.94)	0.68 (0.59-0.87)	0.326
Modified Rankin score, median (IQR)	2.0 (1.0-3.0)	1.50 (1.0-3.0)	2.50 (1.0-3.0)	0.531
CTM-3, median (IQR)	83.3 (77.8-100.0)	77.8 (72.2-100.0)	88.9 (77.8-100.0)	0.640
Re-hospitalization, N (%)	1 (4.2)	0 (0)	1 (10.0)	0.333
ED visit, N (%)	2 (8.7)	2 (12.5)	0 (0)	1.000

** Wilcoxon rank-sum test for continuous variables; Fisher’s exact test for categorical variables.

PCP = Primary care provider; PHQ-8 = Patient Health Questionnaire-8; IQR = Interquartile range; EQ5D = EuroQOL-5-D; CTM-3 = Care Transition Measure-3; ED = Emergency Department.

Overall persistence with discharge medication regimens was 88% (22 of 26), and was similar in both groups (intervention 87.5% and control 88.9%). By medication class, persistence was also high, ranging from 83% for warfarin, 95% for antihypertensives and lipid-lowering medications, and 100% for antiplatelet therapy and diabetes medications.

### Discussion and conclusion

In this study, we pilot-tested and refined a medication coaching script, assessed content validity of the script and questionnaires, and obtained participants’ evaluation of the intervention. We determined that this approach was feasible, and participants expressed a high degree of satisfaction with the information provided by the coach. The medication coaching calls were designed to assess medication knowledge of medications and triage individual questions about medications and the participant’s stroke, per the script. The burden of attempted contacts was reasonable, with a median number of 2 calls to reach members of the intervention group. The 3-month follow-up calls were more challenging because patients moved, changed phone numbers, and experienced major life changes associated with having a stroke. Calling at different times of day often was the key to reaching participants. Other helpful procedures were to determine whether the phone number provided at enrollment is for a pre-paid cell phone and to obtain multiple phone numbers for other relatives when possible. Most importantly, contacting patients and/or proxies requires persistent efforts.

Using a telephone intervention was practical for our stroke population because patients treated at our institution reside in a wide geographic area, making home visits restrictive. Telephone interventions have been used for post-discharge interventions for medication management in multiple settings. For example, patients discharged from a general medical service received a follow-up phone call by a pharmacist 2 days after discharge [17]. Patients randomized to the intervention (n = 110; controls n = 111) were significantly more satisfied with discharge instructions, had medication-related problems solved during the intervention, and had fewer Emergency Department visits (10% vs. 24% in the controls) [17]. A study of 123 patients over age 50 with varying underlying diseases utilized pharmacist follow-up for medication management and found that the intervention led to significantly greater resolution of medication and health-related problems than the control group [21]. Rather than having pharmacists make the calls (as in the above examples), we had a coach who could triage questions to the pharmacist or stroke nurse and supply their answers to the participants. The pharmacist and nurse provided personalized information that could be put in writing for the patient and the primary care provider to keep as a reference. We believe using a coach rather than a pharmacist or nurse for calls may be more economical, although we have not assessed cost-effectiveness in this small study.

Medication non-persistence is associated with poorer outcomes, greater likelihood of re-hospitalization and increased mortality [22,23]. Although this study was not designed to show an effect on medication persistence due to the emphasis on feasibility and refinement of the medication coaching script, medication persistence was excellent in this small study. The Preventing Recurrence of Thromboembolic Events through Coordinated Treatment (PROTECT) program, which included multiple materials for patients at discharge and contact by study nurses 2 to 4 weeks after discharge, showed excellent persistence of antiplatelet therapies, statins, and blood pressure medications in stroke patients at 3 months [24]. Similarly, a pharmacist call for a small cohort of Medicare beneficiaries in Texas showed that persistence with medications was not significantly improved, although the intervention was associated with other measurable benefits, such as resolution of medication and health related questions [21]. The feasibility of medication coaching is currently being tested in Scotland in a randomized controlled trial of 60 patients with stroke to determine the impact on medication adherence [25].

We also examined the impact of early post-discharge medication coaching on medication knowledge and adherence to follow-up appointments. Knowledge related to why medications are taken, how to refill them, and what to do if stroke symptoms recur was excellent in both groups (Table 3), perhaps because of the educational materials all participants received. However, we found that participants in the intervention group were more likely to keep their primary care provider appointments (93.8%) than those in the control group (60%). A key role of the coach is encouraging patients and caregivers to keep medical appointments which is important for preventing hospital readmissions [5], and reducing the risk of stroke and death, especially in patients with hypertension [26].

Short hospital stays following a transient ischemic attack or stroke make in-hospital visits for educating patients about risk factors, medications and post-discharge self-care a challenge. A call from a trained coach just after discharge, when patients are reorienting themselves to home, can provide answers to questions about their stroke and their medications that they may not have thought of during their stay. The coach can also reinforce the crucial messages for prevention of recurrent stroke provided by hospital staff and the transition coach prior to discharge.

Our pilot study was limited because these patients were younger than the average stroke patient, and only those discharged home were enrolled, which limits generalizability. As such, these patients had relatively mild, i.e. non-disabling strokes (Table 1), but these are also the patients who may gain the most from targeted interventions to improve adherence to secondary prevention. We included patients with TIAs in the intervention as well, because these patients have a similar risk of recurrent stroke, and guideline recommendations are the same for both stroke and TIA [27]. Other limitations are the non-random allocation of the intervention and the modification of medication coaching script based on patient feedback. Thus, the script in its final form was administered to 14 of the 19 participants in the intervention group. Most importantly, the small cohort size limits conclusions regarding 3-month outcomes.

## Conclusions

This pilot study demonstrates that educating stroke patients using pharmacy and nurse consultation, via a trained coach represents a feasible and potentially vital tool in early supported discharge after stroke. Contacting patients by phone soon after discharge is a convenient and relatively inexpensive way to provide assistance to patients and caregivers. Larger studies will help determine the full impact and cost-effectiveness of this intervention on medication persistence and other outcomes, such as readmissions and potentially avoidable admissions.

## Competing interests

Dr. Bushnell receives research salary support from NIH/NINDS K02 NS058760, but this funded research is not related in any way to the study presented in this manuscript. Dr. Peterson received salary support from the Agency for Health Care Research Quality, the funding source for this study. None of the other coauthors have any competing interests to disclose.

## Authors’ contributions

ES performed the coaching in the intervention group, provided insights into the refinement of the coaching script, and drafted the manuscript. LZ participated in the design of the study, and wrote sections of the manuscript. LW performed the outcome interviews and participated in the drafting of the manuscript. WP designed and performed the statistical analysis and wrote a portion of the manuscript. DO helped design the study, provided answers to coaching questions, and edited drafts of the manuscript. EP helped design the study, provided oversight to the project, and edited the manuscript. CB guided the design of the study, provided supervision for the conduct of patient enrollment, and the edited each draft of the manuscript. All authors read and approved the final manuscript.

## Pre-publication history

The pre-publication history for this paper can be accessed here:

http://www.biomedcentral.com/1471-2458/12/549/prepub
